# Graphene/Zirconia Composites for Components in Solid Oxide Fuel Cells: Microstructure and Electrical Conductivity

**DOI:** 10.3390/nano15171314

**Published:** 2025-08-26

**Authors:** Francisco J. Coto-Ruiz, Ana de la Cruz-Blanco, Rocío Moriche, Ana Morales-Rodríguez, Rosalía Poyato

**Affiliations:** 1 Instituto de Ciencia de Materiales de Sevilla (ICMS), CSIC-Universidad de Sevilla, Avda. Américo Vespucio 49, 41092 Sevilla, Spain; fj.coto@csic.es (F.J.C.-R.); anadebla@alum.us.es (A.d.l.C.-B.); 2 Departamento de Física de la Materia Condensada, Instituto de Ciencia de Materiales de Sevilla (ICMS), CSIC-Universidad de Sevilla, Avd. Reina Mercedes s/n, 41080 Sevilla, Spain; rmoriche@us.es (R.M.); amr@us.es (A.M.-R.)

**Keywords:** graphene, zirconia, spark plasma sintering, microstructure, electrical conductivity

## Abstract

In this paper, 8 mol% yttria cubic stabilized zirconia (8YCSZ) composites with reduced graphene oxide (rGO) contents up to 10 vol% were consolidated by spark plasma sintering (SPS) at two different temperatures with the aim of evaluating the relationship of their electrical properties with the graphene content, the rGO crystallinity, and the microstructural features. Successful in situ reduction of GO was accomplished during SPS, and highly densified composites with homogeneous rGO distribution, even at the highest contents, were obtained. The electrical properties were analyzed using impedance spectroscopy. Measurements were taken up to 700 °C, revealing an inductive response for the composites with 5 and 10 vol% rGO and a capacitive response for the composites with 1 and 2.5 vol% rGO. The results indicate that, along with the ionic conduction typical of zirconia, there are additional polarization mechanisms associated with the presence of graphene at ceramic grain boundaries that substantially modify the impedance response. A minor electronic conductivity contribution was identified in the composites below the percolation threshold. These characteristics make the 8YCSZ composites promising candidates for application as SOFC components, as ceramic interconnects when the graphene content is above the percolation threshold, or as electrolytes when the graphene content is below this limit.

## 1. Introduction

Nowadays, the progressive increase in energy demand, together with the need to reduce polluting emissions, makes it necessary to look for stable, sustainable, and efficient energy systems with high performance and minimal polluting emissions. An interesting alternative to conventional power generation systems is fuel cell technology [1], which is focused on electrochemical systems, in which the chemical energy stored in a fuel is converted into direct current electricity. In these systems, only pure water steam is generated as a by-product, eliminating the generation of polluting agents.

Solid oxide fuel cells (SOFCs) stand out among other types of fuel cells due to their high efficiency, low CO_2_ emission, and fuel flexibility compared to other fuel cells [2]. In general, these cells incorporate oxygen vacancies containing perovskite oxides or fluorites as oxide–ion electrolytes that promote the transport of oxygen through these vacancies. The most used electrolyte in SOFC is 8 mol% yttria cubic stabilized zirconia (8YCSZ) due to its high conductivity performance and high stability of chemical and mechanical resistances in a wide range of temperatures and different atmospheres [3]. However, its high operation temperature poses an issue to SOFC durability, and different approaches in order to reduce 8YCSZ’s operating temperature for this application have been proposed during recent years. These strategies include optimization of the fabrication process, thickness reduction, and, more recently, 8YCSZ modification with additives, fillers, or dopants [4]. Anodes and cathodes with zirconia-based compositions have been pursued as Ni-yttria stabilized zirconia (YSZ) cermets for anodes or strontium-doped lanthanum manganite (LSM)–YSZ for cathodes, with the aim of avoiding thermal stresses at the electrolyte–electrode interfaces, arising from different thermal expansion coefficients (TECs) of the SOFC component layers, which could result in failure of the cell [5,6]. Furthermore, it has been pointed out that the contact between metal interconnects and cell electrodes is one of the most relevant points for enhancing the stack output performance of planar SOFCs [6]. The use of zirconia-based interconnects would promote the TEC match and chemical compatibility between adjoining parts of the cell and minimizes thermal stresses during device start-up and shutdown [7].

Recent progress in ceramic/graphene composites has revealed that these materials are promising candidates for applications in energy conversion and storage, additive manufacturing technologies, biomedicine, and aerospace and structural engineering [8,9,10]. In recent years, these ceramic composites have been proposed as potential SOFC components, with the aim of improving overall cell performance [11,12,13,14,15,16,17]. Specifically, composite materials with an 8YCSZ matrix and different graphene-based nanostructures as filler have been proposed as potential electrolyte [15,16], anode [11], or ceramic interconnects for SOFCs [15,16,17], depending on the graphene content. In a study by Marinha and Belmonte [15], composites with an 8YCSZ matrix and graphene nanoplatelets (GNP) were deemed promising electrolytes for oxygen transport membranes, presenting a cross-flow of electronic and ionic charge carriers in the presence of a gradient of oxygen partial pressure. The introduction of a certain electronic conductivity, much lower than the ionic conductivity, in the SOFC electrolyte has been shown to be beneficial in increasing the performance of electrodes [14,18], since the electronic conductivity in the electrolyte can extend the active points for electrochemical reactions to take place. These authors suggested that the electrical anisotropy shown by these materials could be tailored near the percolation limit to promote oxygen ion flow through a preferred direction perpendicular to the electronic current [15]. More recently, Glukharev et al. [16] have shown the evolution of electrical conductivity from completely ionic to a mixed ionic–electronic mechanism upon the addition of a reduced amount of reduced graphene oxide. The 8YCSZ composite with 2 wt% rGO presented mixed electronic–ionic conductivity in the 20–800 °C temperature range, which is much lower than the current operating range (600–1000 °C). A few studies [15,16,17] also claimed that 8YCSZ/graphene composites with high graphene contents could be suitable for use as interconnects in SOFCs since they fit several of the requirements that have been shown to be necessary for the materials used in this component, such as high electrical conductivity, chemical, structural and phase stability at operating temperature of 800–1000 °C, gas tightness for both oxygen and hydrogen, matching TEC with 8YCSZ-based electrodes and electrolytes, and moderate mechanical strength combined with outstanding resistance to creep [2]. Gómez-Gómez et al. [17] showed 8YCSZ composites with GNP to be highly tolerant to crack propagation with improved toughness while exhibiting significantly enhanced thermal conductivity and anisotropic TEC. Therefore, these composites could contribute to the enhancement of the performance of SOFCs under demanding operating conditions through a notably improved mechanical stability of the system and the efficiency of the directional heat transfer processes.

The key factors in the optimization of the electrical and mechanical properties of ceramic composites with graphene-based nanostructures are the specific surface area and the number of layers of the filler and its dispersion in the ceramic matrix [19]. Other relevant factors that could be tailored through accurate control of the processing and sintering conditions are the crystallinity of graphene and the interfacial characteristics of ceramic–graphene [20,21,22,23]. Muñoz-Ferreiro et al. [22] showed how the significant enhancement of the few-layer graphene crystallinity achieved during the sintering process resulted in optimum results in terms of electrical conductivity, with the improvement being more remarkable when the sintering temperature increases. The formation at the interface of a C-O-Zr bond was pointed out by Zeng et al. [20] as being responsible for the improved fracture toughness observed in zirconia composites, with rGO obtained by in situ reduction during the sintering process, compared to composites with pre-reduced GO. The extraordinary toughness obtained in Si_3_N_4_ composites with in situ reduced GO was related [21] to a possible strong chemical bonding at the ceramic–graphene interface. To the best of our knowledge, the scarce works that have studied the electrochemical properties of 8YCSZ composites with graphene to date [14,15,16] did not explore the effect of processing and sintering conditions on the microstructure and electrical performance of the composites. Furthermore, only one work studied a wide range of graphene contents [15].

Therefore, the objective of this work is to establish the relationship of the electrical properties of graphene/8YCSZ composites with the graphene content, the crystallinity of the rGO, and the microstructural features, and to elucidate whether these composites could be suitable candidates for SOFC components. To this end, composite powders with GO contents ranging from 1 to 10 vol% were spark plasma sintered at two different temperatures. The in situ reduction of GO during the sintering process and the crystallinity of rGO were analyzed by Raman spectroscopy. The microstructure and crystallographic phases of the materials were characterized by SEM and XRD. Finally, the electrical properties were analyzed by impedance spectroscopy from room temperature up to 700 °C in the directions parallel (σ_||_) and perpendicular (σ_⊥_) to the SPS pressing axis to account for any electrical anisotropy.

## 2. Materials and Methods

### 2.1. Processing and Sintering of Composite Powders

Commercial GO and 8 mol% yttria cubic stabilized zirconia (8YCSZ) powders were utilized as the initial materials. The thickness and lateral dimensions of GO were 2–3 nm and ~7 μm, respectively, (N002-PDE, Angstron Materials, Dayton, OH, USA), and the particle size of 8YCSZ was 52 nm (TZ-8YS, Tosoh Corporation, Tokyo, Japan). The GO powder was bath-sonicated (Sonorex Digitec DT 255, Bandelin, Germany) in isopropyl alcohol for 20 min. Next, 8YCSZ powder was added to the GO suspension in different quantities to prepare composite powders with 1, 2.5, 5, and 10 vol% GO. The slurries were sonicated and dried on a hot plate. Spark plasma sintering of the composite powders was performed at 1300 and 1350 °C for 7 min, applying 75 MPa pressure and 100 and 50 °C/min heating and cooling ramps (SPS model 515 S, Dr. Sinter, Inc., Kanagawa, Japan, Functional Characterization Service, Centro de Investigación, Tecnología e Innovación de la Universidad de Sevilla, CITIUS). In a previous study on tetragonal zirconia with few-layer graphene [22], we showed that a difference of only 50 °C in the sintering temperature resulted in improved graphene crystallinity and electrical conductivity. Thus, in the present work, the effect of close sintering temperatures on the electrical conductivity is also explored. The sintering process was carried out in vacuum, so the reduction of GO was expected to occur in situ during the process [8,23]. Furthermore, monolithic 8YCSZ ceramics were also sintered at 1300 and 1350 °C as reference materials. Details about the powder preparation and sintering process can be found elsewhere [23].

### 2.2. Characterization of Composite Powders and Sintered Specimens

Raman spectra were acquired using a LabRam HR800 spectrometer (Horiba Jobin Yvon, Kyoto, Japan). X-ray diffraction (XRD) patterns were obtained with the diffractometer model D8 Advance A25 (Bruker Co., Billerica, MA, USA). Archimedes’ method was used to obtain the density of the materials. Details about the acquisition of Raman spectra and XRD patterns, and density calculations can be found elsewhere [23].

The size distribution and morphology of the ceramic grains were observed by scanning electron microscopy (SEM, FEI-Teneo, FEI, Hillsboro, OR, USA). The distribution of rGO in the 8YCSZ matrix was observed using the same microscope. Back-scattered electron (BSE) imaging was used for analyzing the distribution of the rGO throughout the matrix in cross-section (c.s.) polished surfaces. Thermal etching was carried out for 20 min at a temperature of 100 °C below the sintering temperature to characterize the morphology of the ceramic grains, as described elsewhere [23].

Electrical characterization was approached by impedance spectroscopy (Agilent 4294A, Agilent Technologies, Santa Clara, CA, USA), with a signal amplitude of 50 mV and a frequency sweep of 40 Hz to 15 MHz. Electrical measurements were carried out up to 700 °C in an atmosphere of argon, in directions parallel (σ_||_) and perpendicular (σ_⊥_) to the pressing axis of the SPS. Two electrode configurations were prepared as described elsewhere [23]. ZView^®^ software (version 4) was used to model the impedance spectra. An equivalent circuit (EC) consisting of a series of two resistances, R, in parallel with constant phase elements, CPE, (two R-CPEs) was used to model the impedance data acquired at temperatures up to 300–350 °C, while for temperatures above this value, an EC consisting of an R-CPE in series with another resistance was used. The values of total electrical conductivity were obtained using the equation

(1) $$\sigma =\frac{1}{R_{T}}\frac{h}{A}$$

where R_T_ is the total resistance obtained, A is the sample area, and h is the thickness between electrodes. The activation energy, E_a_, was calculated from the slope of linear regression fits of Arrhenius conductivity plots.

## 3. Results

### 3.1. Microstructural Study

**Figure 1:**
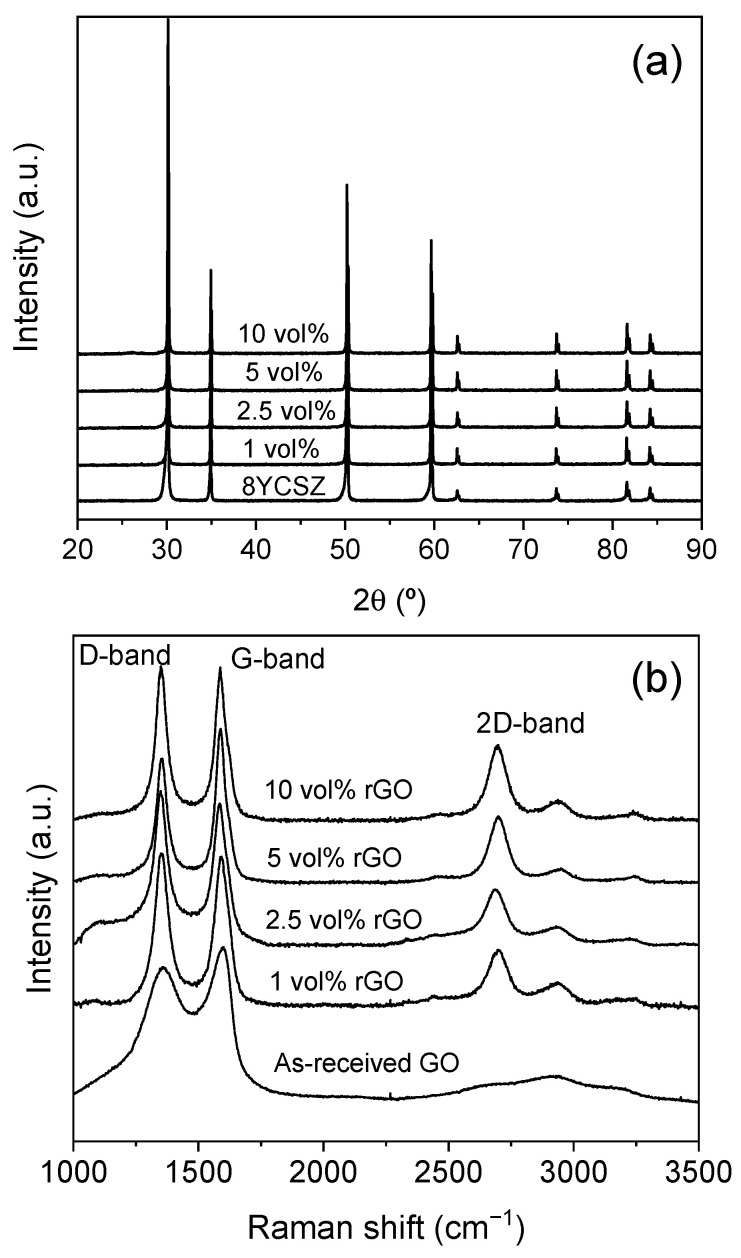
(**a**) XRD patterns and (**b**) Raman spectra of the materials sintered at 1300 °C. The Raman spectrum recorded on the as-received GO is included in (**b**) in order to establish the effect of the sintering process.

Semi-quantitative XRD analysis performed on the sintered monolithic ceramics and composites at the two different temperatures reveals that the main phase in all of them was the cubic zirconia phase, with PDF file 03-0640 (Figure 1a and Figure S1a in Supplementary Information). The in situ reduction of the GO during SPS was analyzed by Raman spectroscopy. Figure 1b and Figure S1b show the Raman spectra of the as-received GO powder and the sintered composites at 1300 and 1350 °C.

A remarkable change in the spectra acquired on the composites after sintering is observed compared to that acquired on GO nanosheets. In the case of GO, broad D and G bands at ~1350 and 1585 cm^−1^, respectively, with a broad shoulder between them (1500 cm^−1^), are observed in the first-order region (1000–2000 cm^−1^). Previous studies [23,24] have revealed that the observed G band is, in fact, an apparent G band (G_app_) formed by the convolution of the G peak and the defect-related D’ peak. Moreover, the broad feature at ~1500 cm^−1^ corresponds to the D’’ peak, related to defective graphite-related materials [22,24,25]. In the second-order region (2250–3500 cm^−1^), no clear peaks are observed, but some bumps corresponding to the convolution of the 2D peak, together with three other different bands related to graphitic materials, are found. In the spectra from the sintered composites, a clear sharpening of the D and Gapp bands is observed. Furthermore, the shoulder at ~1500 cm^−1^ has disappeared. Thus, the relative intensities of the defect-related D, D’, and D’’ peaks with respect to the G peak decrease significantly after the sintering process, revealing that the presence of disorder in the graphene structure and the amorphous carbonaceous phase is remarkably lower than in the as-received GO material. This points to the achievement of partial restoration of the graphene structure during SPS [22,23,26]. Additionally, the appearance of a 2D band as a well-defined band is observed at ~2695 cm^−1^ with a remarkable intensity, which has previously been related to the reduction of GO [27]. Thus, it can be concluded from the present Raman analysis that GO has been successfully reduced in situ during the SPS process. No significant differences are observed between the spectra of the sintered composites at 1300 or 1350 °C, so for both temperatures, the in situ reduction of GO is achieved.

**Figure 2:**
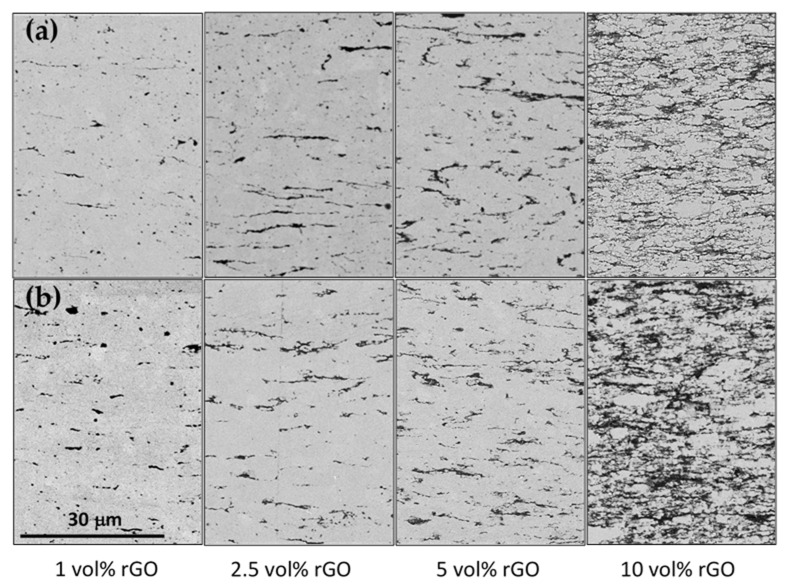
BSE-SEM images of the polished surface of the sintered composites at (**a**) 1300 and (**b**) 1350 °C.

Figure 2 shows the distribution of rGO (dark phase) in the matrix (light phase), revealing a homogenous distribution of rGO, even at the highest contents. The rGO nanosheets appear preferentially oriented along their ab plane, with this plane perpendicular to the applied pressure during sintering, leading to a highly anisotropic microstructure, as previously reported for similar composites [15,17,22,23,28]. No effect of the SPS temperature is observed on the rGO distribution within the matrix. A slight difference in the rGO distribution can be observed between the composites with 10 vol% rGO, with a denser rGO network in the one sintered at 1350 °C. It is likely that this composite presents a slightly higher rGO content due to ceramic powder losses during composite powder processing, as previously reported [28].

**Figure 3:**
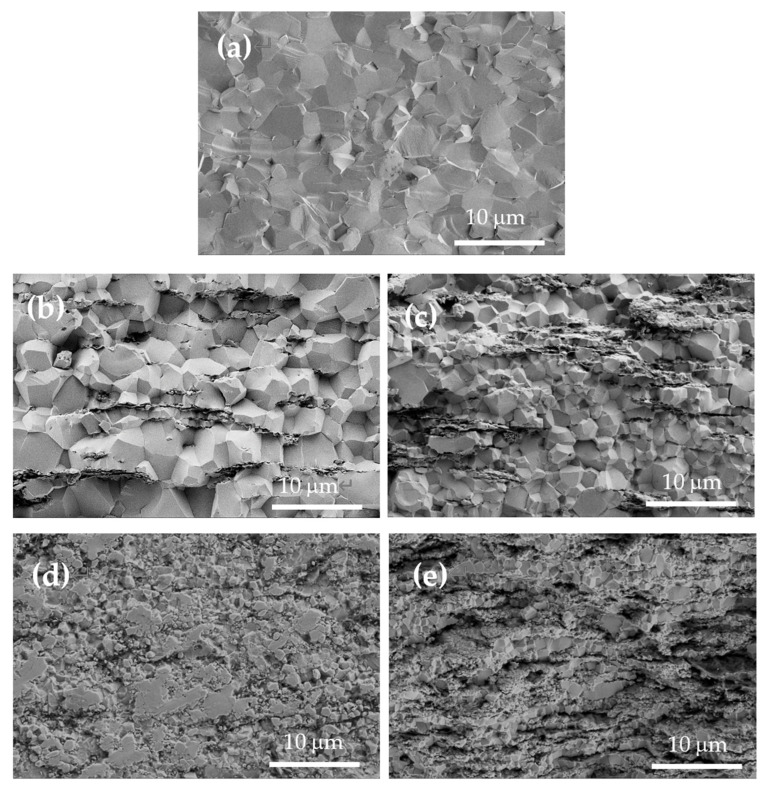
SEM images of the fracture surface of the sintered materials at 1300 °C, revealing intergranular fractures in the composites: 8YCSZ ceramic (**a**), and composites with 1 (**b**), 2.5 (**c**), 5 (**d**), and 10 (**e**) vol% rGO.

Transgranular fractures are observed mainly in the monolithic zirconia ceramics, while intergranular areas also appear in the composites (Figure 3 and Figures S2 and S3 in Supplementary Information). In a study by Flaureau et al. [29], it was observed that intergranular was the main fracture profile in 8YCSZ ceramics at grain sizes lower than 200 nm, and then transgranular fractures started to appear once the grain size reached this value. These authors suggested that samples with transgranular fractures showed higher quality grain boundaries than samples with intergranular fractures. Thus, the presence of some areas of intergranular fractures in the composites could be indicative of a decrease in the grain boundary quality related to the location of the graphene layers between the ceramic grains. However, it could also be related to the lower ceramic grain size achieved in the composites, especially in those with a higher vol% rGO (Table 1). The higher presence of transgranular fractures observed in the composites with lower rGO vol% sintered at 1350 °C would also be a consequence of their grain size, similar to monolithic zirconia (~3 μm).

The rGO nanosheets are distributed along the zirconia grain boundaries as thin, long, and slightly wavy sheets. An intimate cohesion between the ceramic grains and the rGO nanosheets is observed, since the nanosheets barely protrude on the fracture surfaces. Thus, there is no notable extraction of the nanosheets during the fracture. It is worth noting that ceramic grains sandwiched between close rGO nanosheets have a smaller size in comparison to other grains.

**Figure 4:**
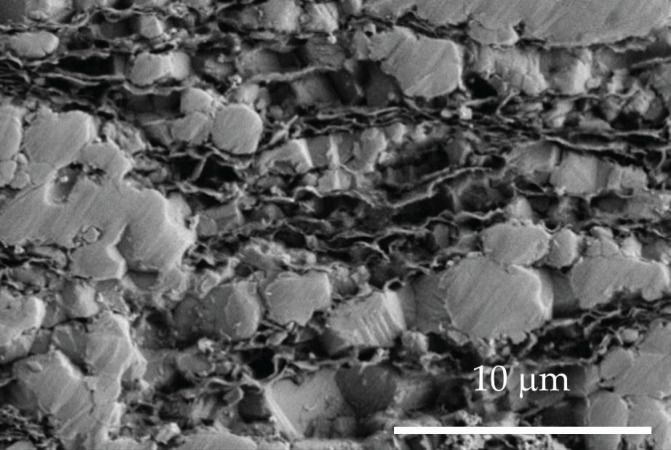
SEM image of the polished surface of the composite with 10 vol% rGO sintered at 1350 °C, showing microcavities between graphene nanosheets.

The full densification obtained for monolithic ceramics (Table 1) is consistent with the previously published results for spark plasma sintered 8YCSZ ceramics prepared under similar SPS conditions [15,29,30]. The SPS technique has been reported to allow the achievement of relative densities in this material higher than those of conventional sintering [31,32]. High relative density was also obtained for both sintering temperatures for composites with rGO contents up to 5 vol% (Table 1), as previously reported for similar composites [15,16,26]. However, a notable decrease in relative density is found for the composites with 10 vol% rGO. Since porosity was not observed in the ceramic matrix, this could be attributed to the presence of microcavities between the graphene sheets, as has been previously reported for ceramic composites with few-layer graphene [28]. Although these microcavities are present in the composites with all rGO graphene contents, their effect on density is more remarkable for the composites with higher rGO vol%. A representative SEM image of the polished surface of the composite with 10 vol% rGO sintered at 1350 °C, revealing some of these microcavities, is presented in Figure 4.

An increase in grain size is observed with rising SPS temperature in both the monolithic zirconia and the composites (Table 1). Rounded grains are observed in all materials, despite the sintering temperature. The grain sizes obtained for the monolithic zirconia are slightly higher than the values published by Flaureau et al. [29] and Rajeswari et al. [30], who reported a grain size of ~1.2 μm for an SPS temperature of 1300 °C. When incorporating rGO into the ceramic matrix, a grain size reduction is observed, with a remarkable drop in grain size for the composite with 10 vol% rGO sintered at 1300 °C and the composites with 5 and 10 vol% rGO sintered at 1350 °C (Table 1). As mentioned above, the ceramic grains sandwiched between close rGO nanosheets present a smaller size. Thus, since in the composites with 10 vol% rGO, there is a great amount of graphene nanosheets excellently distributed throughout the matrix (Figure 2), which would restrict the ceramic grain growth and the densification, the overall effect on the ceramic grain size is more remarkable.

### 3.2. Electrical Properties

**Figure 5:**
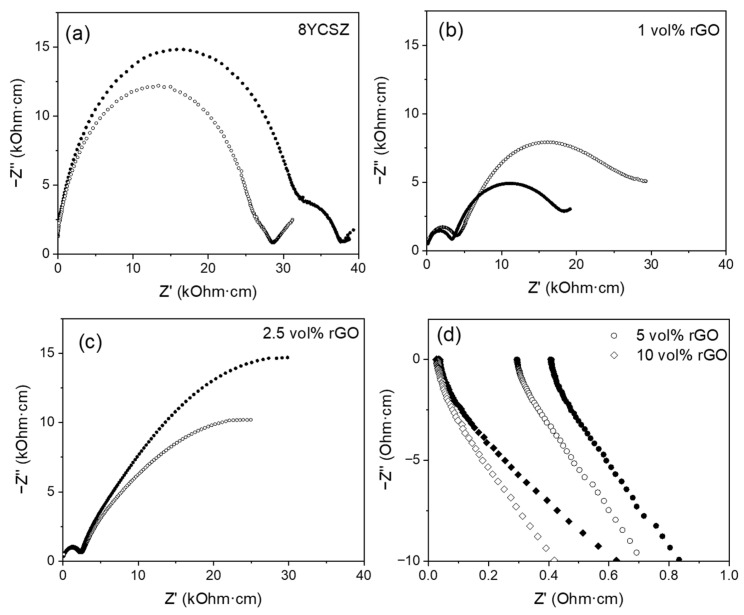
Impedance spectra acquired at 250 °C in configuration σ_⊥_ for (**a**) the monolithic ceramic and (**b**–**d**) the composites with (**b**) 1 vol%, (**c**) 2.5 vol%, and (**d**) 5 and 10 vol% rGO. Solid symbols: sintered at 1300 °C, open symbols: sintered at 1350 °C.

**Figure 6:**
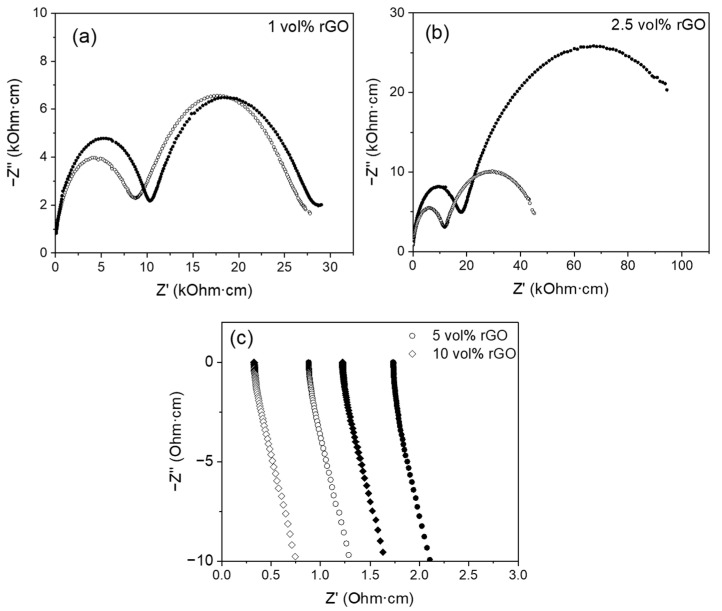
Impedance spectra acquired at 250 °C in the configuration σ_||_ for the composites with (**a**) 1 vol%, (**b**) 2.5 vol%, and (**c**) 5 and 10 vol% rGO. Solid symbols: sintered at 1300 °C, open symbols: sintered at 1350 °C.

The impedance response of the composites is very different depending on their rGO content (Figure 5 and Figure 6). The composites with 1 and 2.5 vol% rGO present a capacitive response from ~200 °C, similar to that of monolithic ceramic, and their impedance spectrum is formed by a succession of arcs. On the contrary, the composites with 5 and 10 vol% rGO present an inductive response from room temperature. This behavior has been previously observed by other authors in similar composites [15]. This change in the impedance response is a consequence of the formation of a percolation network of the graphene sheets that occurs for a percentage between 2.5 and 5 vol% rGO, so that once this threshold is exceeded, conduction is produced by electronic conduction through the percolated graphene network.

Figure 5a–c and Figure 6a,b present representative impedance spectra acquired at 250 °C in the configurations σ_⊥_ and σ_||_, respectively, on the 8YCSZ ceramic and the composites with 1 and 2.5 vol% rGO sintered at 1300 and 1350 °C. The spectra corresponding to the monolithic zirconia are characterized by two arcs at high and low frequency and the beginning of a third arc at the lowest frequencies. Associated capacitances of ~10^−11^–10^−12^ and ~10^−8^–10^−9^ F were obtained for the high- and low-frequency arcs, respectively, regardless of the sintering temperature, using an equivalent circuit consisting of two R-CPEs. These values allow the identification of these mechanisms as the bulk and grain boundary ionic conductivity for zirconia, in accordance with the literature [15]. The feature at low frequency can be related to the oxygen exchange at the platinum electrodes.

In the case of the composites, the impedance spectra at 250 °C show two arcs, and it is at higher temperatures that the beginning of a third arc is observed. Therefore, the spectra of the composites also present two arcs at high and low frequencies and the beginning of a third one at the lowest frequencies (related to oxygen exchange at the electrodes). However, a fundamental qualitative difference is observed in comparison to monolithic ceramics in terms of the size ratio between the arcs at high and low frequencies. While in the monolithic 8YCSZ, the arc at high frequency (bulk) is much larger than the one at low frequency (grain boundary, GB), in the composites, it is the latter that is the largest. Also, a difference between the composites with 1 and 2.5 vol% rGO is observed, with a much larger low-frequency arc observed in the composites with 2.5 vol%.

A change in the ratio between the arcs at high and low frequency has been previously reported for similar systems [11,15,16]. In research by Marinha et al. [15], the appearance of the impedance spectrum for an 8YCSZ composite with 7 vol% GNP is similar to that shown in the present work for the composites with low rGO content (1 and 2.5 vol% rGO). However, these authors associated the large arc on the right part of the spectrum with the electrode response and located the ionic grain boundary conductivity signal as convolved between the arcs, associated with the bulk ionic conductivity and the electrode response. Furthermore, in a study by Glukharev et al. [16], a remarkable change in the size ratio between the arcs at high and low frequency was also found when comparing the impedance spectra from 8YCSZ composites with 0.5 and 1 wt% rGO with the spectrum corresponding to the monolithic zirconia. These authors identified these mechanisms as the bulk and grain boundary ionic conductivity for zirconia and related the slight decrease in total conductivity to the rGO presence at the GB, blocking ion carriers’ movement. In a study by Hudelja et al. [11] on 8YCSZ composites with carbon nanofibers (CNFs), the contribution of the grain boundary resistivity to the overall response increased with higher CNF doping. This was related to CNF accumulation at the GB areas, which would block the routes for ionic conduction across and/or along the grain boundaries.

Differences are also observed when comparing the spectra acquired in the two measurement configurations (σ_⊥_ and σ_||_). The difference in size between the two arcs is much more marked in the spectra acquired in the configuration σ_⊥_ (Figure 5). In this configuration, the largest arc appears very elongated, deformed, to some extent, and incomplete. This effect is more remarkable by increasing the percentage from 1 to 2.5 vol%, since in this case, it is only possible to record half of the arc in the acquisition frequency range. In contrast, in the σ_||_ configuration, the arcs appear more rounded, although they have not been able to be acquired completely in the composites with 2.5 vol% rGO (Figure 6).

The associated capacitances obtained for the high-frequency (C_HF_) and low-frequency (C_LF_) arcs from the fittings carried out with the same equivalent circuit as that used for 8YCSZ ceramics (Table 2 and Table 3) also present remarkable differences compared to the monolithic ceramic. Moreover, significant differences are also observed between the capacitances obtained for the two measurement configurations.

In the σ_||_ configuration, associated capacitances of ~10^−11^ F were obtained for the high-frequency arc in composites with 1 and 2.5 vol% rGO sintered at both temperatures (Table 2). As in the monolithic zirconia, this mechanism is identified with bulk ionic conduction. However, the associated capacitances obtained for the low-frequency arc are only consistent with those of monolithic zirconia (~10^−8^–10^−9^ F) in the composite with 1 vol% rGO sintered at 1300 °C. Capacitances in the range of ~10^−7^–10^−8^ F were found for this arc in the rest of the composites. In the σ_⊥_ configuration, higher associated capacitances (~1–2 orders of magnitude) than in the 8YCSZ ceramic were obtained for both arcs in all the composites except the one with 1 vol% rGO sintered at 1300 °C (Table 3), being the effect more remarkable for the low-frequency arc in the composites sintered at 1350 °C. Therefore, the two arcs in the impedance spectra can be exclusively related to the bulk and grain boundary ionic conduction only in the composite with 1 vol% rGO sintered at 1300 °C. In the rest of the composites, our results indicate that, along with the ionic conduction by bulk and grain boundaries typical of zirconia, there are additional mechanisms associated with the presence of graphene at the grain boundaries that substantially modify the impedance response, resulting in an enlarged low-frequency arc and increased capacitances. These additional mechanisms have a more remarkable effect in the composites with 2.5 vol% rGO as a consequence of the higher presence of graphene at the ceramic grain boundaries, resulting in a significantly larger low-frequency arc in these composites, compared to the composites with 1 vol% rGO. Moreover, these mechanisms have a more significant effect in the σ_⊥_ configuration, since in this case, the capacitances associated with both impedance arcs are affected. Further studies are required in order to identify the origin and character of these mechanisms. Consequently, the deconvolved bulk and grain boundary responses are not determined for the composites with 1 and 2.5 vol% rGO in the present study. Only the total sample conductivity is analyzed and compared with the values obtained for the composites with 5 and 10 vol% rGO, which presented an inductive behavior, since for these composites, although it is not possible to deconvolute the bulk and grain boundary responses, total conductivity can be determined.

**Figure 7:**
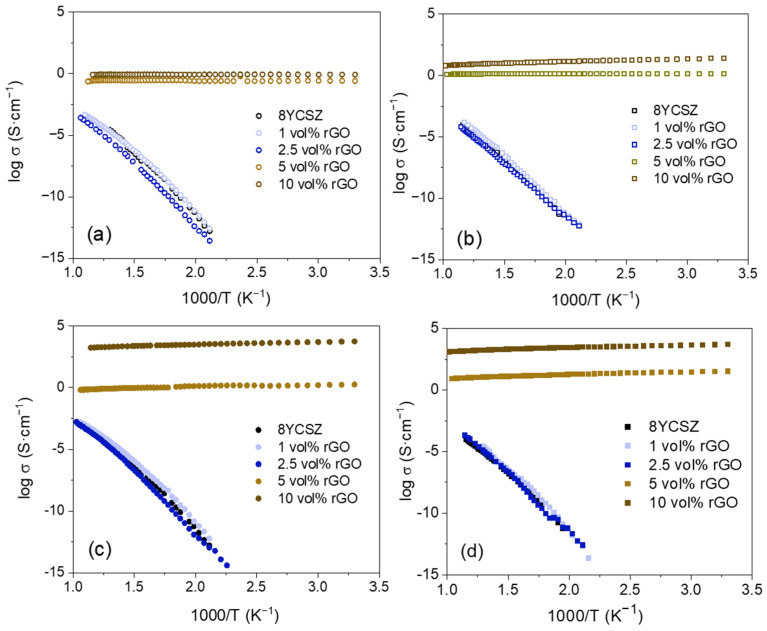
Arrhenius plots of the total electrical conductivity of the composites sintered at (**a**,**c**) 1300 °C, (**b**,**d**) 1350 °C: (**a**,**b**) configuration σ_||_, (**c**,**d**) configuration σ_⊥_.

Accordingly, Figure 7 presents the Arrhenius plots of the total electrical conductivity of the sintered monolithic ceramics and the composites at 1300 and 1350 °C, respectively, characterized in both configurations σ_||_ and σ_⊥_. The electrical conductivities of the composites with 5 and 10 vol% rGO are several orders of magnitude higher than the obtained ones for the composites with lower rGO contents, regardless of the sintering temperature. Previous studies have reported that for composites with graphene contents above the percolation threshold, conduction takes place by means of electronic conductivity through the graphene network [15,33]. The extremely high conductivity of graphene [34] and the interconnections between the nanosheets when exceeding the percolation threshold result in the remarkable increase in conductivity observed for these composites.

For the composites above the percolation threshold, the conductivities present an almost flat curve in the whole temperature range, with a certain negative slope in the most conductive composites, revealing a metallic-type behavior. The higher values acquired for the σ_⊥_ configuration are a consequence of the preferred alignment of the nanosheets in the direction perpendicular to the applied pressure during SPS, since, when acquiring in this configuration, charge transport occurs along the main graphene ab plane, as previously reported [15,28]. In the σ_||_ configuration (Figure 7a,b), conductivity is very similar for all the composites regardless of the rGO content. Nevertheless, a significant increase in conductivity is observed in the σ_⊥_ configuration (Figure 7c,d) when the amount of graphene is increased from 5 to 10 vol%. In this configuration, the increase in rGO content results in a notable increase in the number of interconnections between the main ab planes of the graphene sheets, promoting the subsequent rise in conductivity.

An effect of the SPS temperature on the conductivities of the composites is observed above the percolation threshold, with higher values obtained for the composites sintered at 1350 °C. Previous studies have shown that an increase in the sintering temperature of ceramic composites with graphene fillers can promote enhanced electrical conductivity by decreasing the amount of structural defects and amorphous carbon in the graphene structure [22]. In the present study, no significant differences were observed in the Raman spectra of the sintered composites at 1300 and 1350 °C (Figure 1b and Figure S1b) that could indicate differences in the number of defects. However, the higher conductivities of the sintered composites at 1350 °C reveal that an optimized restoration of the graphene structure has taken place, thanks to an enhanced GO reduction process when sintering at the highest temperature. These composites with enhanced electronic conductivity could be suitable candidates for SOFC ceramic interconnects due to their marked electrical anisotropy and the possibility of minimizing the thermal stresses in the cell due to the thermal expansion coefficient matching with 8YCSZ-based electrodes and electrolytes. Nevertheless, further efforts should be made to achieve full densification in order to fulfill the gas-tightness required for these SOFC components and to prove their properties in both extremely oxidizing and reducing atmospheres.

Regarding the composites with 1 and 2.5 vol% rGO, they show activation energies similar to the ones presented by the monolithic ceramics (0.9–1 eV) for the two explored sintering temperatures. These values, typical of ionic conduction through oxygen vacancies in zirconia, indicate that the ionic conductivity in the matrix is dominating the electrical behavior.

The composites with 1 vol% rGO sintered at 1300 and 1350 °C present a total conductivity slightly higher than the obtained one for the monolithic ceramics (enlargements of the Arrhenius plots of the composites with 1 and 2.5 vol% rGO are presented in Figures S4 and S5 in Supplementary Information to better illustrate the behavior of these composites). The highest conductivities were obtained for the configuration σ_⊥_, despite the enlarged arc at low frequency observed in the impedance spectra (Figure 5b and Figure 6a) that we have related to the existence of additional polarization mechanisms. A decrease in total resistivity has also been reported for 8YCSZ composites with 1 wt% carbon nanofibers [11]. This was related to the proximity of this CNF content to the percolation threshold, since an increasing amount of zirconia grains would be short-circuiting with highly conducting CNF. Thus, in the composites in the present study, most probably, there are already percolation paths through which certain electronic conductivity takes place, promoting an increase in the total conductivity. These paths are most likely formed in the composites with 2.5 vol% rGO, closer to the percolation threshold, and in the σ_⊥_ configuration, in which the charge transport takes place through the ab plane of the aligned nanosheets. In the composite with 2.5 vol% rGO sintered at 1300 °C, a decrease in the total conductivity is found in comparison with the monolithic ceramic and the composite with 1 vol% rGO. In this case, although conduction paths have also formed, since this sample would be closer to the percolation limit than the 1 vol% composites, the prominent increase in the arc at low frequency (Figure 5c and Figure 6b) causes the total conductivity to decrease. As a consequence of the preferential alignment of the rGO nanosheets, the total conductivity in this composite almost reaches the value of the 8YCSZ ceramic for the σ_⊥_ configuration. An increase in the total conductivity is found when sintering the composite with 2.5 vol% rGO at 1350 °C. For this composite, although the shape of the impedance spectra is the same as in the composite sintered at 1300 °C, i.e., the low-frequency arc is much larger than the high-frequency one, the entire spectrum is smaller than for the composite sintered at the lowest temperature. This indicates that, although graphene certainly has an effect on the low-frequency arc (more remarkable in the composites with 2.5 vol% rGO), generating new polarization phenomena, as we have already mentioned, the optimized crystallinity that has been achieved by a better reduction of the GO by sintering at a higher temperature results in an enhanced charge transport through the percolation paths that have been already formed at these low graphene contents.

The electronic conductivity is not high enough to modify the activation energy in any of the analyzed composites with a low rGO content, so our analysis indicates that these materials are mixed ionic–electronic conductors (MIECs) with a very minor electronic contribution. Although further studies are needed to quantitatively extract the ionic and minor electronic contribution, and studies in both oxidizing and reducing atmospheres should be performed, these materials could prove to be suitable candidates for use as a MIEC electrolyte for SOFCs with improved performance, since the minor electronic conductivity in the electrolyte could extend the active points for electrochemical reactions to take place [14,18].

## 4. Conclusions

Highly dense 8YCSZ composites with a homogeneous distribution of preferentially aligned in situ reduced graphene oxide (rGO) were obtained by spark plasma sintering for rGO contents up to 10 vol%. The rGO nanosheets were disposed with their ab plane perpendicular to the uniaxial pressure during SPS. As a consequence of the presence of microcavities between the graphene sheets, a drop in relative density and grain size in the composites with the highest rGO contents was observed.

The composites with 5 and 10 vol% rGO presented an inductive response from room temperature, revealing a conductivity with metallic-type behavior due to electronic conduction through the percolated graphene network. Higher conductivity values were acquired for the σ_⊥_ configuration as a consequence of the preferential alignment of the nanosheets. An increase in the conductivities of these composites above the percolation threshold was observed when sintering at 1350 °C as a result of the enhanced restoration of the structure of graphene due to the improved GO reduction process achieved when sintering at the highest temperature.

A capacitive response similar to that of monolithic ceramic was observed from ~200 °C in the compounds with 1 and 2.5 vol% rGO. However, the qualitative differences observed in terms of the size ratio between the arcs at high and low frequencies in comparison to the monolithic ceramics reveal that, along with the ionic conduction by bulk and grain boundaries typical of zirconia, there are additional polarization mechanisms associated with the presence of graphene at the grain boundaries that substantially modify the impedance response. Slightly higher conductivities than in the monolithic ceramics were achieved in the composites with 1 vol% rGO, despite the enlarged arc at low frequency observed in the impedance spectra, as a result of the formation of percolation paths through which a certain electronic conductivity takes place. In the case of the composites with 2.5 vol% rGO, only when sintering at 1350 °C is an increase in the total conductivity found, thanks to the optimized graphene crystallinity achieved at the highest temperature, which results in an enhanced charge transport through the percolation paths that have already formed at these graphene contents. Although the electronic conductivity is not high enough to modify the activation energy in any of the analyzed composites, these materials are identified as mixed ionic–electronic conductors (MIECs) with a very minor electronic contribution. These 8YCSZ composites appear to be promising candidates for application as SOFC ceramic interconnects when the graphene vol% is above the percolation threshold, or as SOFC electrolytes when the graphene vol% is below this limit.

## Figures and Tables

**Table 1:** Relative density (ρ_r_ (%)), mean grain size (<*d*> (μm)), and aspect ratio (*A.R.*) with standard deviation (σ_<*d*>_ (μm) and σ_A.R._) of the studied materials.

rGO Vol%	T_sint_ (°C)	ρ_r_ (%)	<*d*> (μm)	σ_<*d*>_ (μm)	*A.R.*	σ_A.R._
0	1300	100	2.2	1.2	1.5	0.4
1		100	1.5	0.8	1.6	0.8
2.5		99	1.3	1.4	1.6	0.6
5		98	1.2	1.0	1.6	0.6
10		94	0.5	0.1	1.6	0.5
0	1350	100	3.7	2.2	1.5	0.4
1		100	2.9	1.6	1.5	0.3
2.5		98	3.1	2.3	1.5	0.4
5		98	1.1	0.9	1.5	0.4
10		94	0.7	0.5	1.6	0.5

**Table 2:** The associated capacitances obtained from the simulation using an equivalent circuit consisting of two R-CPEs * for the composites with 1 and 2.5 vol% rGO in the σ_||_ configuration.

rGO Vol%	T_sint_ (°C)	C_HF_ (F)	C_LF_ (F)
1	1300	~10^−11^	~10^−8^–10^−9^
2.5		~10^−11^	~10^−7^–10^−8^
1	1350	~10^−11^	~10^−8^
2.5		~10^−11^	~10^−8^

* In the temperature range of 200–350 °C.

**Table 3:** The associated capacitances obtained from the simulation using an equivalent circuit consisting of two R-CPEs * for the composites with 1 and 2.5 vol% rGO in the σ_⊥_ configuration.

rGO Vol%	T_sint_ (°C)	C_HF_ (F)	C_LF_ (F)
1	1300	~10^−11^	~10^−8^–10^−9^
2.5		~10^−10^	~10^−7^
1	1350	~10^−10^–10^−11^	~10^−7^–10^−8^
2.5		~10^−10^–10^−11^	~10^−6^–10^−7^

* In the temperature range of 200–350 °C.

## Data Availability

Data will be made available upon request.

## Supplementary Materials

The following supporting information can be downloaded at: https://www.mdpi.com/article/10.3390/nano15171314/s1, Figure S1: (a) X-ray diffraction patterns and (b) Raman spectra of the materials sintered at 1350 °C. The Raman spectrum recorded on the as-received GO has been included in (b) in order to establish the effect of the sintering process; Figure S2: SEM images of the fracture surface of the materials sintered at 1350 °C: 8YCSZ ceramic (a), and composites with 1 (b), 2.5 (b), 5 (c) and 10 (d) vol% rGO; Figure S3: SEM images of the fracture surface of the composites with (a) 1 and (b) 2.5 vol% rGO sintered at 1300 °C, revealing transgranular fracture; Figure S4: Arrhenius plots of the total electrical conductivity in the temperature range 200-350 °C for the samples below the percolation threshold sintered at 1300 °C: (a) configuration σ_||_ and (b) configuration σ_⊥_; Figure S5: Arrhenius plots of the total electrical conductivity in the temperature range 200-350 °C for the samples below the percolation threshold sintered at 1350 °C: (a) configuration σ_||_ and (b) configuration σ_⊥_.
