# Supplementary material for: Graphene/Zirconia Composites for Components in Solid Oxide Fuel Cells: Microstructure and Electrical Conductivity
Source: Nanomaterials (Basel). 2025 Aug 26;15(17):1314. doi: 10.3390/nano15171314 (PMC12430071; doi:10.3390/nano15171314)
Supplement: Supplementary file 1 [file nanomaterials-15-01314-s001.zip › nanomaterials-3805090-supplementary.pdf]

SUPPLEMENTARY INFORMATION

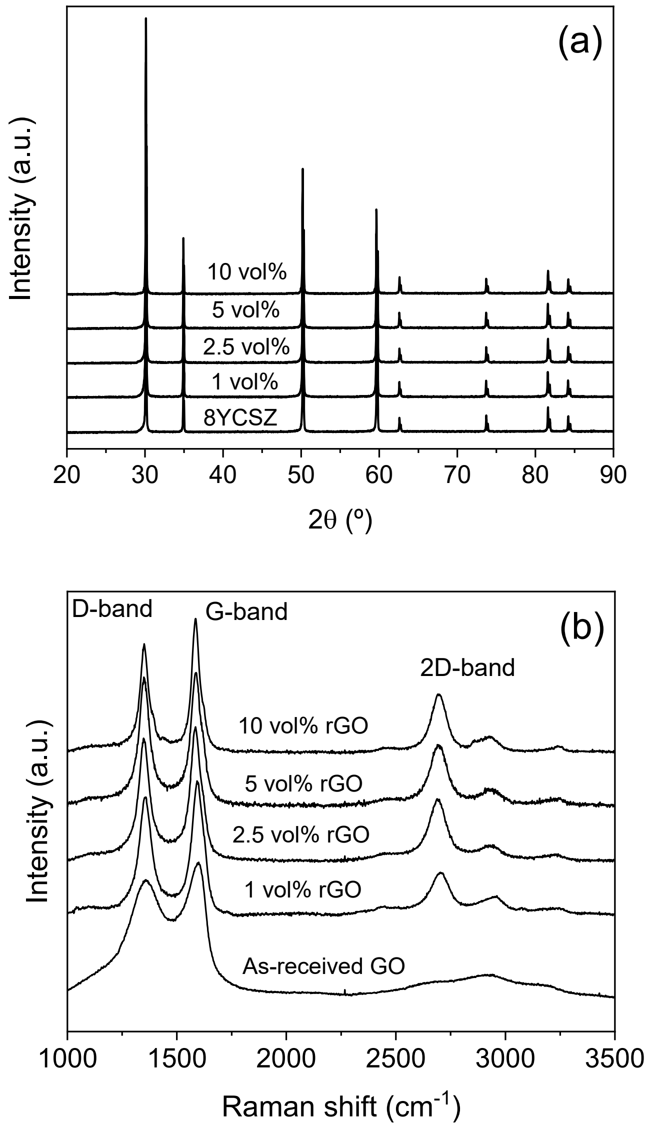

Figure S1: (a) X-ray diffraction patterns and (b) Raman spectra of the materials sintered at 1350 °C. The Raman spectrum recorded on the as-received GO has been included in (b) in order to establish the effect of the sintering process.

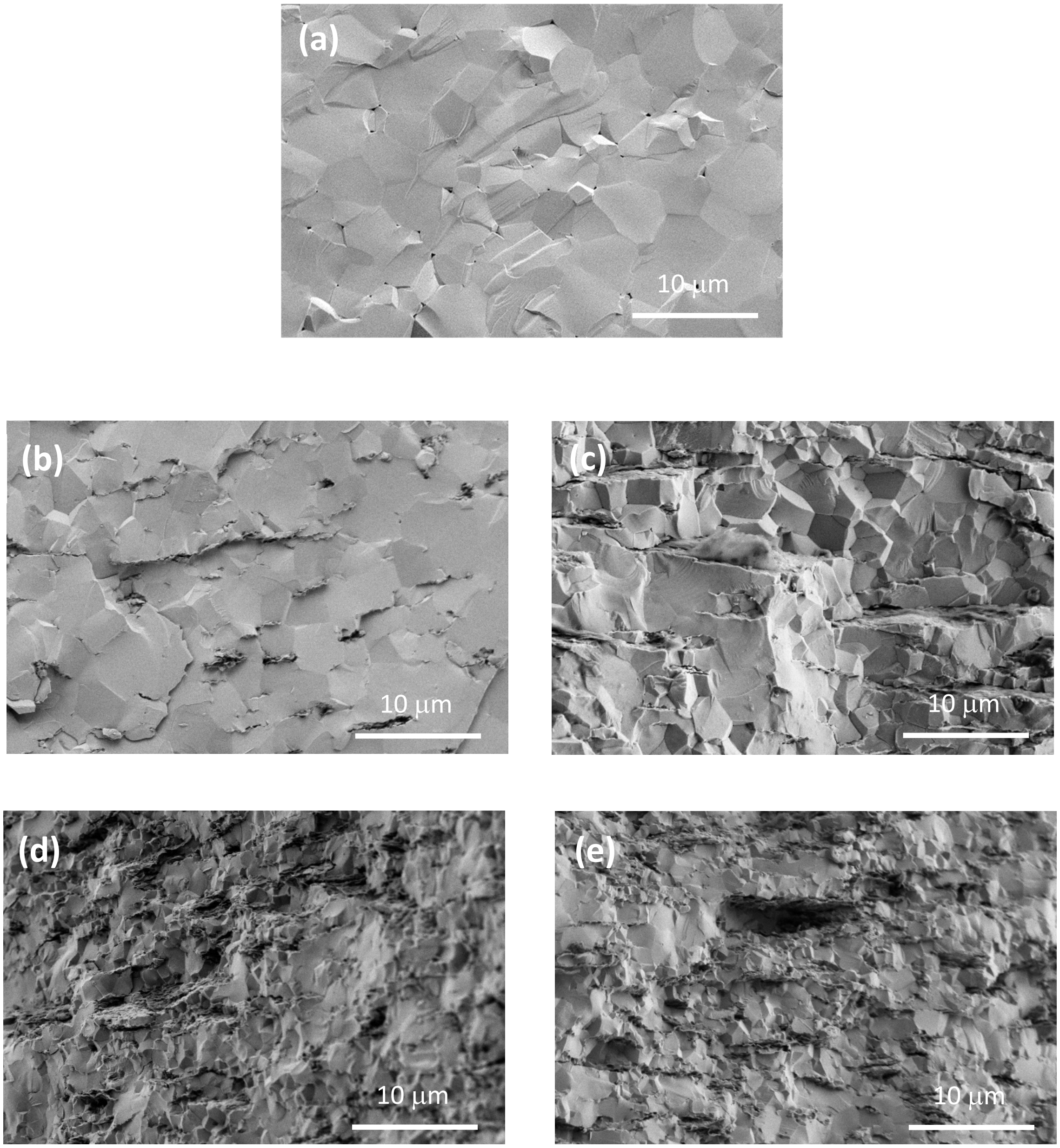

Figure S2: SEM images of the fracture surface of the materials sintered at 1350 °C: 8YCSZ ceramic (a), and composites with 1 (b), 2.5 (b), 5 (c) and 10 (d) vol% rGO.

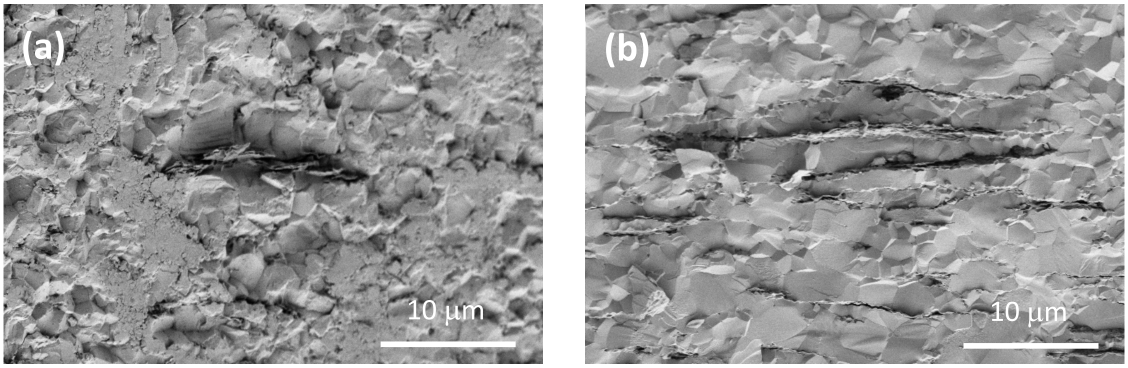

Figure S3: SEM images of the fracture surface of the composites with (a) 1 and (b) 2.5 vol% rGO sintered at 1300 °C, revealing transgranular fracture.

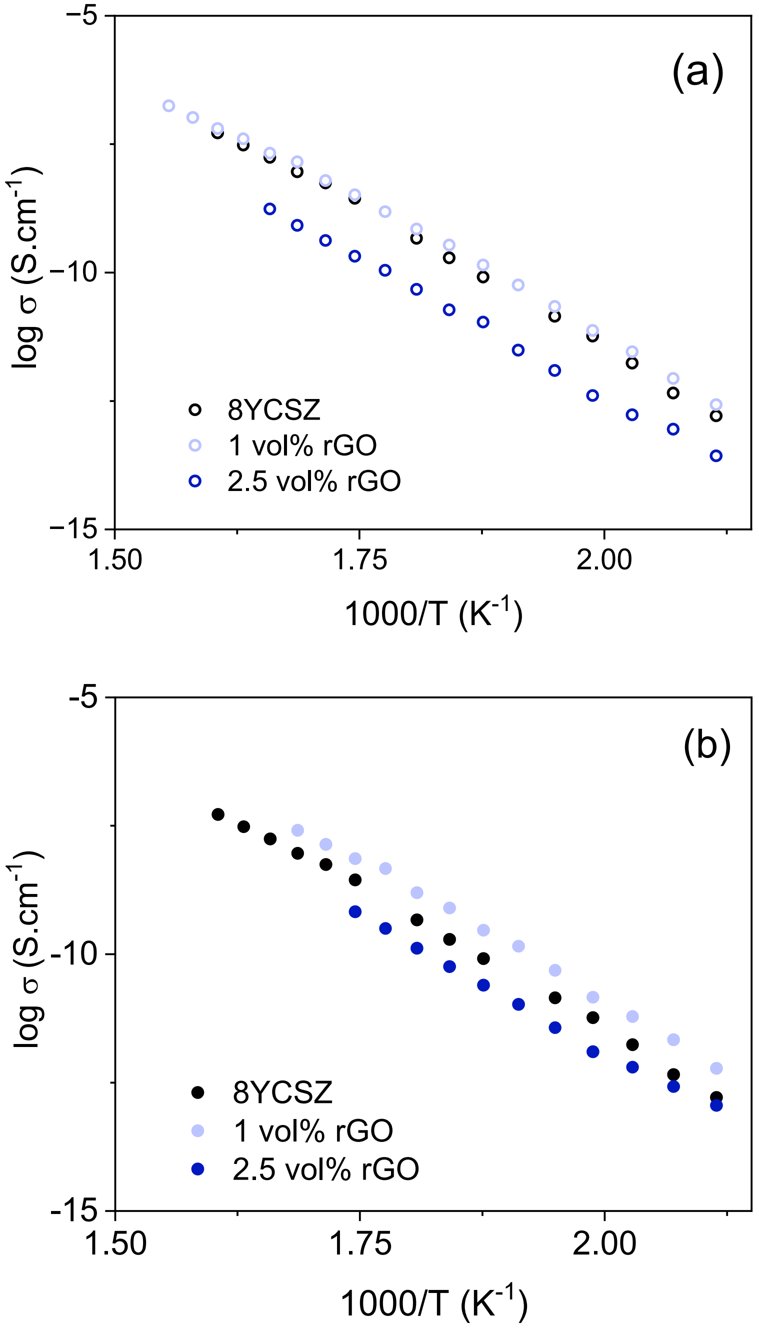

Figure S4: Arrhenius plots of the total electrical conductivity in the temperature range 200-350 °C for the samples below the percolation threshold sintered at 1300 °C: (a) configuration $\sigma_{||}$ and (b) configuration $\sigma_{\perp}$.

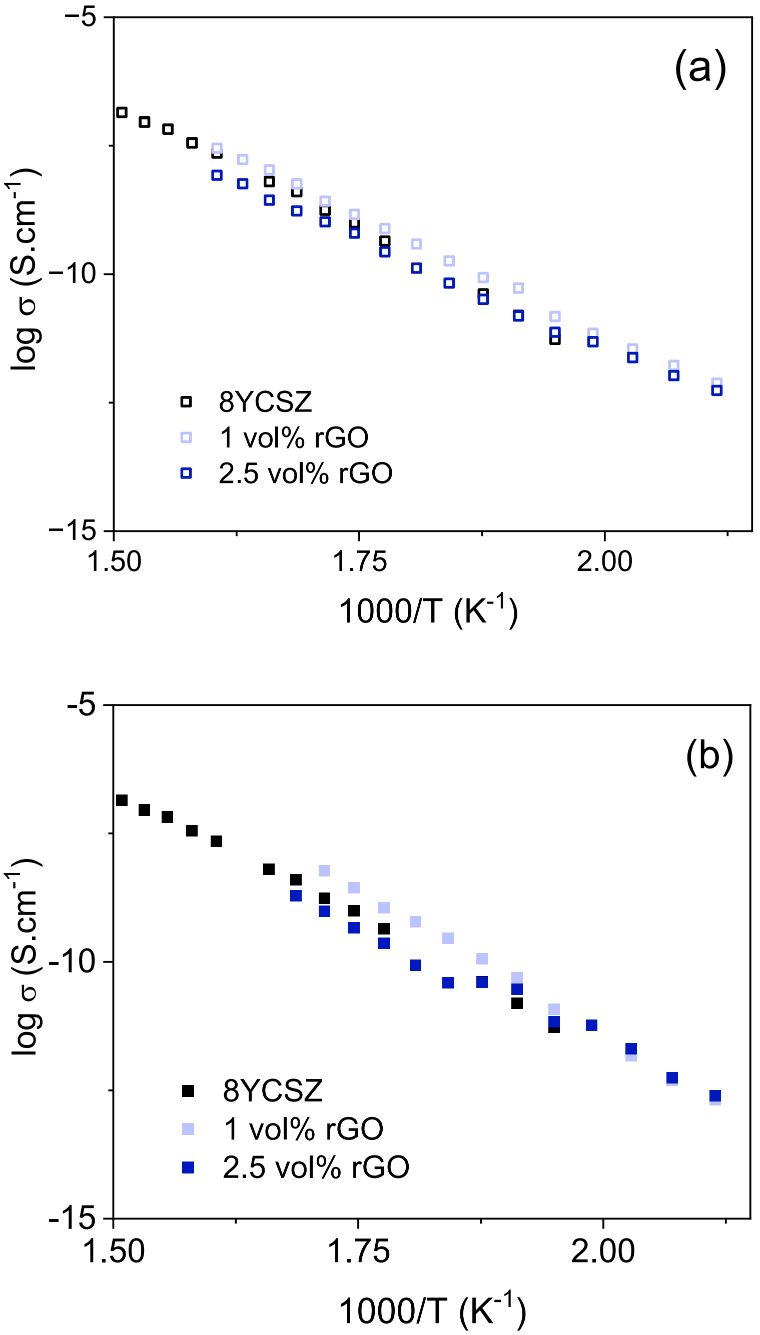

Figure S5: Arrhenius plots of the total electrical conductivity in the temperature range 200-350 °C for the samples below the percolation threshold sintered at 1350 °C: (a) configuration $\sigma_{||}$ and (b) configuration $\sigma_{\perp}$.
